# Noise Driven Evolutionary Waves

**DOI:** 10.1371/journal.pcbi.1002005

**Published:** 2011-03-10

**Authors:** Oskar Hallatschek

**Affiliations:** Biophysics and Evolutionary Dynamics Group, Max–Planck Institute for Dynamics and Self–Organization, Göttingen, Germany; University of Texas at Austin, United States of America

## Abstract

Adaptation in spatially extended populations entails the propagation of evolutionary novelties across habitat ranges. Driven by natural selection, beneficial mutations sweep through the population in a “wave of advance”. The standard model for these traveling waves, due to R. Fisher and A. Kolmogorov, plays an important role in many scientific areas besides evolution, such as ecology, epidemiology, chemical kinetics, and recently even in particle physics. Here, we extend the Fisher–Kolmogorov model to account for mutations that confer an increase in the density of the population, for instance as a result of an improved metabolic efficiency. We show that these mutations invade by the action of random genetic drift, even if the mutations are slightly deleterious. The ensuing class of noise-driven waves are characterized by a wave speed that decreases with increasing population sizes, contrary to conventional Fisher–Kolmogorov waves. When a trade-off exists between density and growth rate, an evolutionary optimal population density can be predicted. Our simulations and analytical results show that genetic drift in conjunction with spatial structure promotes the economical use of limited resources. The simplicity of our model, which lacks any complex interactions between individuals, suggests that noise-induced pattern formation may arise in many complex biological systems including evolution.

## Author Summary

Mutations that increase an organism's fitness are the fuel for biological evolution. When such beneficial mutations enter a spatially extended population, they spread through the population in a “wave of advance”, first described by R. Fisher and A. Kolmogorov. The force driving these traveling waves is Darwinian selection, which favors individuals with higher fitness. Here, we describe a new type of traveling mutant wave that is driven by non-selective forces instead-- namely by random genetic drift, which refers to the randomness in the reproduction process. These noise-driven waves promote the economical use of a limited resource because they occur whenever a mutation increases the growth yield, which refers to the biomass produced per unit of resource. Since a change in growth yield and growth rate often occur together and with opposite signs, we argue that both types of mechanisms will jointly decide over the fate of a novel mutation. We predict that the population evolves towards an evolutionary optimal carrying capacity, at which selective and non-selective forces just balance.

## Introduction

The fact that survival and reproduction are sometimes a matter of luck rather than fitness, has arguably left many traces in the history of evolution [1]–[3]. Random accidents in the reproductive process lead to sampling errors in the chain of generations. When accumulated over time, these sampling errors can cause significant changes in the abundance of genetic variants. This phenomenon, called random genetic drift, can represent a significant hurdle for adaptation [2]. For instance, newly arising beneficial mutations are usually lost by chance and need to occur many times, until they succeed in reaching fixation [4]. More generally, random sampling errors tend to reduce diversity by eliminating rare variants from the gene pool. Spatially extended populations are thereby fragmented into patches in which different genetic variants (alleles) dominate [5]. Allele frequency gradients between patches are maintained by a balance of genetic drift and dispersal [4].

Such spatial structure has important consequences for the process of adaptation. In a spatial setting, novel beneficial mutations occur in one place and need to spread across the habitat to reach fixation [6]. These mutant invasions proceed in the form of waves, first described by R.A. Fisher and A. Kolmogorov et al. in 1937 [7], [8]. Their deterministic analysis of the combined effects of selection and diffusion reveals a characteristic wave speed, which depends on migration and growth rates. Subsequently, it was found that Fisher-Kolmogorov waves appear in most complex systems and control the speed of numerous important dynamical processes, such as chemical reactions [9], bacterial colony growth [10] or epidemic outbreaks [11], [12]. As a result, Fisher-Kolmogorov waves have been investigated not only in biology, but also in chemistry and physics [13]–[15].

An entirely deterministic analysis of traveling waves is incomplete as it neglects genetic drift, which is inevitable in finite systems. Already R.A. Fisher noticed that random fluctuations play an important role in selecting a unique wave speed. The sensitivity of traveling waves to genetic drift started to become fully appreciated when stochastic computer simulations became feasible [16]. This spurred intensive research efforts, in particular in the statistical physics community, to augment the deterministic analysis by random sampling noise [17]. The ensuing stochastic Fisher–Kolmogorov waves are characterized by fluctuating wave fronts and strongly reduced wave speeds. Noise acts as a drag force in these nonlinear systems, with the result that the deterministic wave speed is a threshold that is only approached slowly as population sizes tend to infinity [18]–[20].

Here, we show that random sampling errors can also *drive* traveling waves. We analyze the stochastic mechanism underlying these noise-driven waves and quantify the conditions under which they emerge in complex biological or physical systems. In the context of evolution, noise-driven waves ensue from the competition for a single limited resource in a spatially extended habitat. Importantly, this phenomenon promotes the evolution of the economical use of a limited resource, which has been hypothesized as one of the earliest forms of altruism, already present at the level of microbial biofilms.

Capturing the phenomenon of noise-driven waves requires a fundamental extension of the Fisher-Kolmogorov model for the invasion of mutants. This standard model (and its variants) exclusively deals with mutations that change the growth rate while having no effect on the growth “yield” – that is the biomass produced per unit of resource. The spread of such growth rate mutations is slowed down by noise, as described above. However, a change in growth rate is expected to involve a change in growth yield as well, as several recent studies have advocated on the basis of thermodynamic principles [21], [22]. To account for such a trade-off between growth and yield, we extend the Fisher-Kolmogorov model in a minimal way to be able to describe mutations that change both rate and yield. The resulting model supports noise-driven waves because in the presence of number fluctuations, nearby individuals are related, and all gain from an increase in local density. Noise driven waves, hence, arise from a form of kin selection (a version of group selection), which we quantify using simulations and a novel analytical approach.

### Model

**Figure 1:**
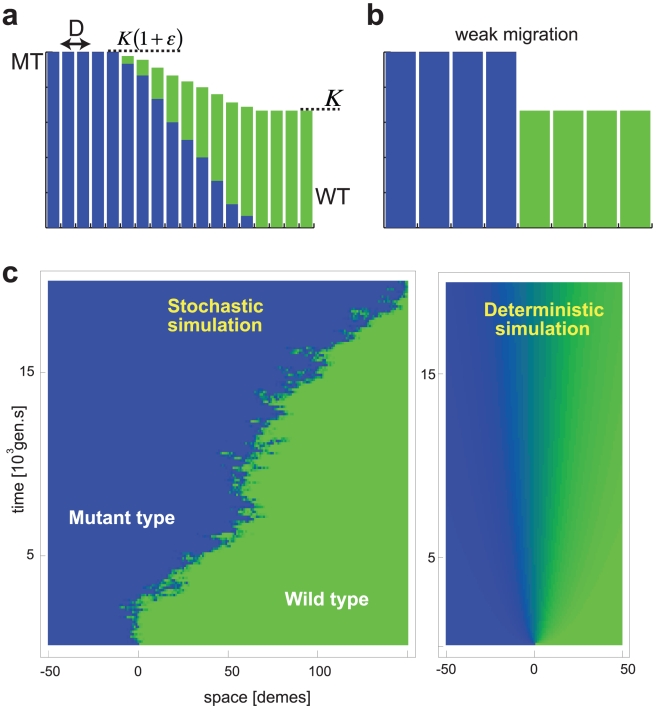
Noise can drive traveling waves. A computer model is used to simulate the competition for a common resource between two species, mutants (blue) and wild type (green). Mutants are assumed to use resources more economically than the wild-type. As a consequence, higher population densities can be sustained in the mutant regions. Yet, mutants are unable to invade the wild-type population unless the randomness in the reproduction process (genetic drift) is implemented in the computer model. a) The spatially extended population is represented by a linear array of local populations, called demes. Individuals migrate between neighboring demes at a rate 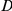 per generation. The population size of the demes ranges from 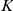 for demes that are occupied by wild-type only to 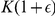 for mutant only demes. Due to the diffusive mixing of both types, the transition from MT to WT occurs in general over more than one deme. b) For very low migration rates, demes are either fixed for the wild-type (WT) or the mutant type (MT), and the transition between both regions is step-like. c) Representative results of stochastic (left) and deterministic (right) simulations with parameters 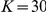, 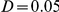 and 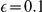. Horizontal and vertical axes represent space and time, respectively. The color shading intermediate between blue (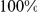 mutants) and green (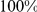 wild-type) indicates the mixture between both types at a given deme. Note that i) mutants invade the wild-type population only in the stochastic simulations, ii) the transition region between mutants and wild type remains stable in the stochastic case but gradually blurs in the deterministic simulations.

Our computer model, illustrated in figure 1, provides the setting for the competition of two types, mutants and wild type, in a spatially extended population. It consists of a linear array of sub-populations, called demes. Individuals have a chance 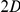 per generation to jump to one of the neighboring demes. The growth of mutants (0) and wild type (1) within a deme from generation 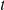 to 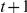 is simulated by the following rule 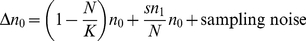
(1)

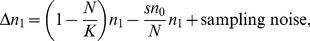
(2)where 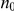 and 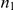 are the numbers of wild type and mutants in generation 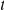, respectively, and 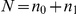 is the total population size of the deme. The first term on each of the right hand sides describes the logistic growth of the deme population 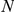: The growth rate declines linearly with increasing population size and vanishes at certain maximal occupancy 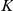. This “carrying capacity” 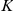 is the equilibrium population size per deme at which resource production and consumption just balance. It represents the population density that the environment can sustain, given the available necessities. The second term on the right hand side of each of the equations (1) and (2) accounts for a small difference 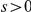 in the growth rate of mutants and wild type. This implements natural selection against the mutant type in a standard way. Notice that we have chosen selection to act on the ratios of both types but not directly on the total deme population 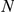: The 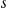-dependent terms in equations (1) and (2) add up to zero. Finally, genetic drift arises in our model from the sampling noise in equations (1, 2), which we generate using standard Wright-Fisher sampling [4].

With constant carrying capacity 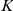, the above model simply represents a discretized version of the standard Fisher-Kolmogorov model. For 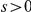, the wild type sweeps through the population in the form of a traveling wave, thereby displacing the mutant type. However, as we demonstrate below, the assumption of a constant carrying capacity has to be relaxed to account for mutations that change the organism's growth yield (biomass produced per unit resource). Therefore, we go beyond the Fisher-Kolmogorov setting and allow for the possibility that the carrying capacity depends on the local composition of the population. Specifically, we assume that a population entirely consisting of mutants has a carrying capacity 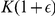 as opposed to 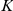 in a purely wild-type population, see Fig. 1a, b. The (small) parameter 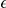 quantifies the strength of the mutation. In a mixed population with mutant frequency 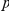, the carrying capacity is assumed to be given by 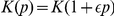.

Biologically, such a frequency dependent carrying capacity arises whenever the mutant type consumes less resource per generation than the wild type (equivalently, whenever mutants produce more biomass per unit of resource). Such yield-mutants will leave more of the limited resource to its immediate neighbors, notwithstanding their identity, with the net-result of an increased carrying capacity. Natural realisations of this scenario are provided by many microbial species that can boost their growth rates by (partially) shifting catabolic substrate flow into less-energy-conserving branches, resulting in lower biomass yields [23]. For instance, yeasts can switch their metabolism from respiration to fermentation plus respiration [24], [25]. Respiration results in higher yield but slower substrate turnover and growth rate. Using fermentation in addition to respiration results in lower yield but higher substrate turnover and growth rate. Mutations with immediate effect on carrying capacity also occur when bacteria compete for space rather than nutrients, as in a tightly packed biofilm [22]. A mutation that reduces slightly the space requirements of a mutant cell will effectively increase the local carrying capacity: A population containing a fraction of mutants will be able to reach higher cell densities than an all wild type population.

As these microbial examples show, a frequency dependent carrying capacity is an important biological alternative when different types compete for the same limited resource (nutrients, water, sunlight, space, etc.). To highlight the novel effects associated with such a frequency dependent carrying capacity, which lies outside the scope of traditional wave models, we begin our analysis by assuming that the growth rates of mutants and wild type are identical. In the second part of the analysis, however, we will assign a growth rate cost (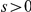) to the mutants because it is quite unlikely that an increase in population density comes without any cost. Indeed, in the case of microbes competing for the same nutrient source, it is predicted that an increase in metabolic efficiency is usually associated with a decreased growth rate [21], [22], [24]. This case of a trade-off [21] between growth rate and yield has received particular attention in the recent literature, and will be discussed in the second part of the analysis.

At first, however, we will investigate the above model assuming 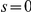 in order to answer the question whether mutations with 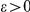 will prevail despite the fact that they lack a direct fitness difference. To this end, we stage a “tug of war” between both types. That is, we assume that, initially, all individuals in one half space (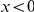) are mutants and the entire population in the other half-space (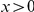) is wild-type. As individuals migrate and reproduce, this initially step-like transition between both types evolves into a more or less smooth interface. Shape and motion of this mixing zone determine whether the mutant invasion will succeed or fail.

## Results

**Figure 2:**
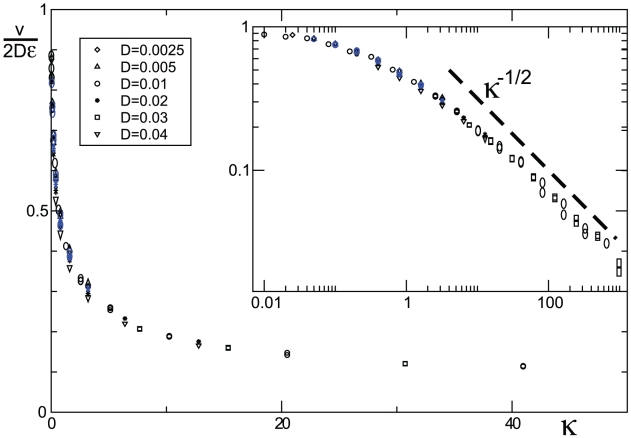
Speed of noise driven mutant invasions. The wave speed 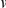 was measured in units of 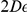 and plotted as a function of the parameter combination 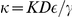. Data sets with different migration rates 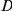 were used to generate this scaling plot, as indicated in the legend box. The effect 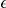 of the mutations was set to 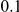 (black symbols) or 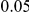 (blue symbols); the variance in offspring number was chosen to be 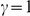. Note that the different data sets collapse onto a single curve. For small 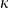, this “master” curve saturates at 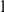 corresponding to a wave speed 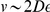. On the double logarithmic scale (inset), the data approaches a straight dashed line for large 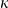, consistent with the predicted asymptotic power law dependence 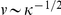.

We find that, in any *finite* population, mutants can invade (only) with the help of local number fluctuations. That is, the interface between mutants and wild-type gradually shifts towards the wild-type region, as in the simulation Fig. 1c (left). The importance of sampling noise can be verified in purely deterministic simulations that neglect genetic drift, see Fig. 1c (right). Note that the transition region between mutants and wild-type remains at a fixed position and merely broadens diffusively over time. To quantify how strongly mutants dominate over wild-type in finite populations, we measured the invasion speed as a function of the model parameters. The simulation results, summarized in Fig. 2, suggest that the invasion dynamics is controlled by a single parameter 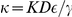, combining carrying capacity 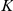, diffusivity 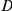, relative increase 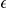 of the carrying capacity of mutants, and the variance 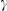 in the offspring number of individuals. The parameter 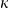 compares the effect of diffusion with the strength of stochastic fluctuations. For large 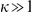, the wave front extends over many demes, and moves slowly with weak front diffusion. For small 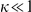, on the other hand, wave fronts are step-like and exhibit strong diffusion. The simulation results in Fig. 2 suggest that the wave speed 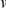 in both regimes can be summarized as 
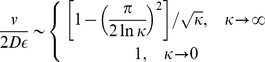
(3)


How can one rationalise the stochastic mechanism underlying these noise driven waves? An intuitive argument can be given for the regime 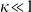, which occurs when the migration rates or local population sizes are small. Then, the flux of migrants is so small compared to the fixation time within a deme, that the transition from wild-type to mutants occurs between two neighboring demes. Hence, the situation usually looks as in Fig. 1b with a step-like interface between wild-type and mutant regions. Under these conditions, the transition region shifts one deme into the wild-type region if a mutant migrates into the first wild-type deme and reaches fixation there. Such events occur at rate 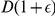 because mutant migrants appear in the wild-type region at a rate 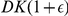, and fix with probability 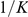. Conversely, the transition region may shift towards the mutant domain if a wild-type becomes established in the first mutant deme. The corresponding transition rate is given by the product of the rate at which wild-type migrants appear in the first mutant deme, 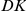, and the fixation probability of a wild-type in mutant demes, 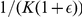. The back and forth stepping of the transition region results in a net speed of 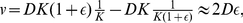
(4)


in agreement with the small 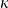 limit of our simulation results. This simple argument shows that the invasion of mutants is made possible by the fact that i) mutants more often attempt to invade wild-type demes than the other way around and ii) that invasion attempts have a higher success probability. Both effects are the result of the larger carrying capacity of mutant demes, and contribute the same amount 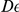 to the average invasion speed.

The situation becomes more complicated when the mixing zone between both types extends over many demes (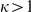), and the wave front is smeared out. Nevertheless, the general case can be treated analytically (see Methods). This is made possible by a nonlinear variable transformation due to E. Hopf and J.D. Cole [26], [27], which converts our model of noise driven waves onto the conventional Fisher–Kolmogorov model with parameters that depend on the noise strength. This exact mapping shows that the combination of migration and stochasticity confers an *effective* growth rate advantage of 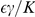 to the mutants. The results for the wave speed in Eq. (3) then follow from the known asymptotic results for noisy Fisher–Kolmogorov waves [19], [28], [29].

**Figure 3:**
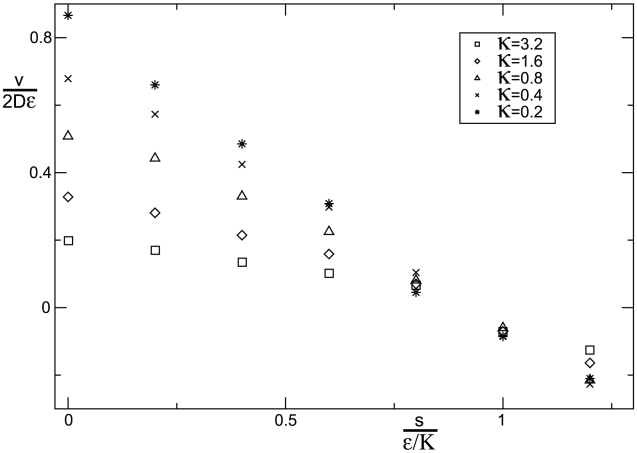
Balance between natural selection and random sampling noise. The wave speed in units of 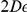 is depicted as a function of a selective disadvantage 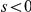 of mutants that increase the carrying capacity by a factor 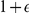. Under these conditions, sampling noise and natural selection act in opposite directions - noise favors mutants, natural selection favors wild type. Note that the mutant population expands (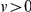) provided that the selective disadvantage 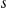 is less than the ratio of 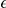 and the carrying capacity 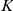. The point at which the speed changes sign defines an evolutionary stable strategy as discussed in the text. The data exhibits a deviation of about 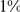 from the predicted point of sign change. This deviation can be lowered by using smaller migration rates (data not shown). Migration rates were set to 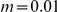 and different values of 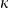 were used, see the legend box.

Due to the noise-induced growth rate advantage, mutants will always out-compete the wild-type population provided both types have equal intrinsic growth rate, or fitness. However, as we discussed earlier, the mutants' ability to increase population densities will usually be associated with growth rate determinant. For heterotrophic organisms, in fact, such a correlation follows from basic thermodynamic principles of ATP production [21], [22], [24]. To account for this trade-off between growth rate and yield [21], we have studied our model for a selective disadvantage 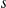 of the mutants. We find both in simulations (Fig. 3) and theory (Methods) that the noise induced excess growth rate (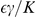) must be larger than the fitness cost (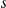) to ensure invasion of the mutants. As a consequence of this “force” balance, we can determine an optimal carrying capacity 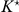, at which mutations are unable to invade. To this end, we assume that relative change 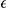 in carrying capacity is linearly related to the relative change 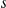 in growth rate 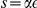, where the number 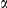 characterizes the evolutionary costs associated with a small change in carrying capacity. We expect such a linear relation to hold at least for small 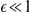. Balancing the evolutionary cost for increasing carrying capacities (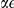) with the noise induced growth rate of mutants (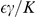) yields 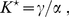
(5)which is the carrying capacity for which mutations with non-zero 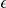 are unable to invade. In the frame work of evolutionary game theory [30], the condition in Eq. (5) is called an evolutionary stable strategy towards which populations are expected to evolve on long evolutionary time scales.

## Discussion

The emergence of an optimal carrying capacity is intriguing because, even though using resources more efficiently seems to be good for the group, it is not clear how resource efficiency could evolve if it implies a fitness cost. The resulting evolutionary dilemma is analogous to the “tragedy of the commons”, a metaphor widely used to describe evolution towards the inefficient use of a common resource [31]. This puzzle is particularly striking in microbial populations that exhibit a wide spectrum of phenotypes between fast growing strains with low efficiency in ATP production and slow growing high efficiency strains [22], [24]. It has been argued that the economical utilisation of resources may be one of the earliest form of altruism, since it is wide-spread already at the level of microbial systems [22]. The emergence of this basic form of cooperation in spatially extended habitats has been observed in individual-based simulations [21], [22], [32], but (to our knowledge) no theoretical account could yet quantify the effect. On the contrary, attempts to describe the spread of mutations using the classical Fisher-Kolmogorov approach, which is based on deterministic reaction diffusion equations, came to the conclusion that density increasing mutations are unable to invade [33], [34].

Our analytical results show that stochasticity is the key difference between the individual based simulations and the deterministic theory. Random genetic drift favors mutations that increase the carrying capacity. It thereby promotes the economical use of a limited resource even if this implies a small growth rate detriment. The strength of this effect crucially depends on the parameter 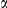, characterizing the trade-off between growth rate and yield. If, for instance, we consider microbes competing for the same nutrient source, we expect that a mutant type that consumes less nutrients will suffer from a comparable reduction in growth rate. In this case, the parameter 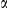 will be on the order of 1 with the consequence that equation (5) predicts a rather small evolutionary stable carrying capacity 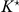. The opposite situation may arise, for instance, when bacteria are competing for space in a dense biofilm. Then, mutant cells would occupy smaller volumes, which could be neutral (or even beneficial) in terms of growth rates. This would imply 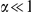 and, because noise would be strong compared to selection, a rather large evolutionary stable carrying capacity 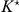. Thus, in systems where the density changing mutations have little effect on relative fitness but large effect on density, the carrying capacity might indeed result from the balance of noise and selection, as predicted by equation (5).

Our study thus provides a predictive null model for the joint evolution of growth rate and yield, which shows that intricate interactions between individuals are not required for the evolution of resource efficiency in spatially extended populations. All that is required is (inevitable) genetic drift in conjunction with spatial structure, which is particularly strong in microbial biofilms. Real biofilms are often characterized by heterogeneous resource distributions, environmental fluctuations, intrinsic instabilities (e.g., finger or sector formation in biofilms), or self-organisation (Touring mechanism), which are beyond our simple null-model. Such spatio-temporal heterogeneities are expected to further increase the levels of genetic drift. Our predictions for the evolutionary optimal carrying capacity should therefore be interpreted as lower bounds for real systems.

The mechanism underlying noise-driven waves can be understood in several ways. Within the theory of “kin selection” [35], which is a special case of group selection [36], one tries to rationalise the advantage of cooperative mutants in terms of an increased relatedness, which makes it more likely that the altruistic benefits are received by conspecifics rather than wild type. From this point of view, genetic drift generates increased relatedness in our model and allows mutants to invade despite a growth rate detriment.

A more direct way of rationalizing the role of noise in our model is provided by our discussion of the regime of low migration rates in the Results section. There, we showed that mutants enjoy a higher diffusion flux into the wild type demes and a higher probability of becoming fixed there. Crucially, these advantages require frequency gradients. If mutants were homogeneously distributed in the habitat, diffusion fluxes and fixation probabilities would be identical for all individuals, independent of their identity. This entirely mixed state, lacking any frequency gradients, is in fact the equilibrium state of our model in the deterministic limit of infinite population sizes (Methods). Consequently, the wave speed of noise-driven waves declines as population sizes tends to infinity. For any finite population size, however, frequency gradients are continually generated by the action of genetic drift. In the scenario of our model, these (random) frequency gradients turn into an advantage for the mutants.

The importance of frequency gradients for noise-driven waves is clarified mathematically in the Methods section. There, we show that the local growth rate of the mutant frequency is proportional to the square of local frequency gradients. These gradients are generated by genetic drift, leading to an effective growth rate advantage of mutants. A similar mathematical structure occurs in certain reaction diffusion models of group selection, which also exhibit growth rates proportional the square of frequency gradients [36]. Barton and Clark in Ref. [36] gave an heuristic explanation of how this mathematical structure could lead to an effective mean growth rate, considering a balanced polymorphism in the limit of small genetic drift. Our exact analysis based on the Cole-Hopf transformation justifies the use of an effective local growth rate and shows that it is given by 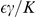, which depends on the carrying capacity 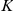, the relative increase 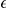 of the mutant carrying capacity, and the variance 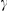 in offspring numbers. (The scaling (not the pre-factor) of our *local* effective growth rate is consistent with the *mean* effective growth rate obtained by Barton and Clark [36].) It is quite remarkable that this effective growth rate and, consequently, the evolutionary stable strategy in equation (5) do not depend on either diffusion constant 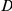 nor the dimensionality, even though migration and population structure are needed for the phenomenon of noise driven waves.

In summary, we have seen that the established Fisher wave model is unable to account for a trade-off between growth rate and yield. To overcome this limitation, we have generalized the Fisher-Kolmogorov wave model such that mutations are allowed that change both the growth rate and the carrying capacity. We found that the extended model exhibits traveling waves that are driven by random sampling errors. The ensuing noise driven waves are described analytically and compared with classical Fisher–Kolmogorov waves. The most striking difference is that the speed of noise driven waves decreases (like a power law) as population sizes tend to infinity, quite in contrast to classical Fisher–Kolmogorov waves. Comparing the strength of the noise-induced driving force with natural selection led us to the prediction of an evolutionary optimal carrying capacity. This implies that random genetic drift promotes the economical use of a limited resource, one of the most basic forms of altruism. We suspect that this mechanism has been acting over long evolutionary times, because it merely rests on random genetic drift in conjunction with spatial structure, which must have been present already in the most ancient microbial systems. In the sense of Wright's shifting balance hypothesis [37], our model describes a mechanism of peak shifts that relies on pure chance rather than selection.

Although our model was formulated with an evolutionary application in mind, its mathematical structure arises in many problems that combine diffusion and interaction of discrete entities. Sampling errors turn into a driving force whenever reaction rates depend on the magnitude of gradients. This occurs, for instance, in problems where the diffusivities depend on population densities [14], or vary among species, which can lead to Turing patterns [38]. Thus, pattern formation by genetic drift may be an important mechanism in many complex systems including biological evolution.

## Methods

### Noise as a driving force

Here, we give an analytic derivation of our result equation (3) for the wave speed of noise driven waves in the absence of any direct selection against the mutant type. Our analysis is based on nonlinear variable transformation that maps the model of noise driven waves to classical Fisher-Kolmogorov waves. The following also discloses the general mathematical conditions, for which noise can act as a driving force in pattern forming systems.

The main text contained a brief intuitive argument for the wave speed under conditions of small migration rates, where the transition between wild-type and mutant demes is step-like, as in Fig. 1b. This weak migration limit was relatively easy to analyze because the state of the system frequently returns to a well-defined initial state (renewal process). Next, we consider the other extreme, in which the dynamics becomes deterministic. As mentisoned in the main text, previous studies as well as our simulations [33], [34] indicate the absence of traveling waves in this deterministic limit, and we would like to explain these observations analytically. The general (and most interesting) stochastic case with intermediate migration rates is treated subsequently by adding the appropriate fluctuations.

In the deterministic limit, the migration of individuals between demes can be approximated by diffusion with diffusivity 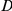. In this framework, the spatially varying population density is described by a field 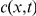 that depends on time 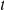 and a continuous deme index 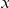. The dynamics of this field is given by a spatial analog of the logistic equation,
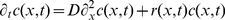
(6)where the local growth rate 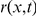 depends on the ratio between total population density and local carrying capacity, and reads 
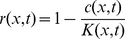
(7)in our units of time. Whereas for small densities 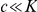, the growth rate equals the linear birth rate 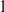 per generation, the growth rate disappears at carrying capacity 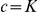, which is a general feature of logistic growth. As discussed in the main text, the carrying capacity 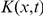 depends on the local frequency 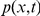 of mutants by virtue of 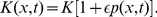
(8)


As mutants and wild-type are subject to the same migration and growth rates, the evolution equation for the mutant density 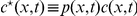 must have the same form as equation (6), 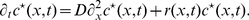
(9)


It is convenient to eliminate 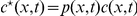 in favor of the frequency 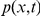 of mutants because 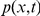 appears in the expression for the carrying capacity, equation (8). After a further substitution 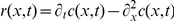 from equation (6), we obtain 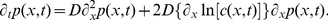
(10)


We seek a solution of equation (6) and equation (10) for a step function initial condition, 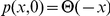.

In the Supporting Text S1, we show that the density closely follows the carrying capacity, provided that the migration rate is small, 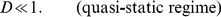
(11)


The assumption becomes exact in the limit 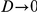 while 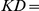
const. In this quasi-static regime, we may substitute 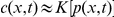 in equation (10) to arrive at a closed equation for 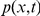,
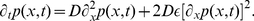
(12)


The nonlinearity proportional to 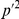 is non-negative everywhere. Neglecting this term might cause serious errors as its integral over the whole space could be large or even divergent. In fact, the nonlinearity 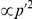 turns out to be a singular perturbation and, thus, the crucial point of equation (12).

Fortunately, this nonlinearity can be removed by a variable transformation due to E. Hopf and J.D. Cole [26], [27]. Thereto, we introduce the new dynamical field 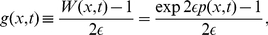
(13)which represents the fraction of mutants to leading order in 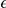, 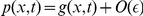. In terms of this new field, equation (12) transforms into a simple diffusion equation 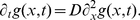
(14)


It is clear that the diffusion equation does not admit traveling wave solutions. Instead, equation (14) with a step function initial condition has a solution of the form 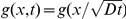, which can be easily found analytically. The form 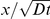 of the scaling variable suggests that the solution describes a front that is slowly broadening due to diffusion. The typical width and position of the front grows as the characteristic length scale 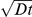 for diffusion. Even though the mean position of the front moves towards the wild-type domain, it does so at an ever decreasing speed. Both observations, front broadening and vanishing front speed, are consistent with the deterministic simulations reported in the main text, Fig. 1c (right). There, we had to conclude that mutants are not able to invade in the deterministic limit.

For 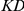 large *but finite*, however, we can no longer neglect sampling errors (genetic drift). The mutant frequency then becomes a stochastic field that fluctuates due to population turnover from generation to generation. These sampling errors, for example generated by Wright-Fisher sampling [4], cause a noise term in the equations (10,12), which reads [39], [40]

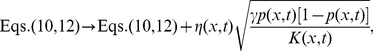
(15)where 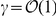 is the variance in offspring number and the stochastic forcing term 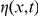 has white noise correlations,
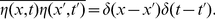
(16)


The square of the amplitude in front of the noise term in equation (15) represents the expected variance in mutant frequency due to the sampling from generation to generation.

Altogether, the *noisy* dynamics of the mutants' frequency is described by 
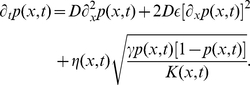
(17)


In contrast to the deterministic case, frequency gradients 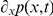 remain finite in the long time limit as they are continuously generated by the noise term. It thus seems reasonable that these fluctuations could turn the gradient squared term into a veritable growth term. How strong will this stochastic driving force be?

It turns out that this effect becomes manifest when we apply the above nonlinear variable transformation to the stochastic differential equation (17). In doing so, one has to appreciate that stochastic differential equations have peculiar transformation rules. These so-called Ito transformation rules result from the fact that, during a short time interval 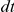, fluctuations have an amplitude proportional to 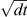 (like a random walk) instead of 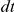. As a consequence, a non-linear variable transformation automatically leads to an additional drift term in the transformed equation, called a “spurious” drift term [41]. The Cole-Hopf transformation (13) therefore results in equation (14) plus a spurious drift term and a noise term. The new drift term has the form of a logistic growth term,
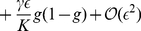
(18)favoring the growth of the mutants. The noise term takes the form 
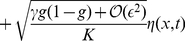
(19)on the right hand side.

The suppressed terms of order 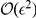 turn out to become of higher order than the displayed terms after the following rescaling 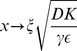
(20)

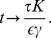
(21)


With these substitutions, the stochastic equation of motion takes the form 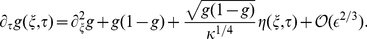
(22)


The suppressed terms are now of higher order, 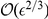, and may be neglected for small 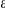. The remaining leading order of equation (22) has the form of a noisy Fisher-Kolmogorov wave equation [7], [8]. The parameter 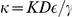, introduced in the main text, represents the effective strength of the noise term. The asymptotic behavior of the wave speed as a function of 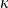 reported in equation (3) and Fig. 2 now follows from known results [7], [8], [19], [28], [29] on the stochastic Fisher-Kolmogorov equation.

Finally, we note that the square gradient nonlinearity in equation (12) was the crucial mathematical structure from which our noise driven waves emerged. It is clear that similar waves arise in any reaction diffusion system of discrete objects provided that the reaction terms contain similar gradient square non-linearities. In these systems, noise turns into a driving force because it randomly creates and maintains gradients, which are absent in the deterministic limit.

### Trade-off between growth rate and yield

As mentioned earlier, there are general reasons to posit a trade-off between growth rates and densities, at least in heterotrophic organisms [21]. This means that mutants that use resources more efficiently (and therefore allow for higher population densities) may suffer from a reduced fitness. To account for this possibility, we have included a selective disadvantage 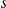 for the mutants; i.e. we assume that the growth rate mutants is by a factor 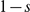, 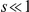 smaller than that of the wild-type, which is 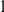 in the chosen units of time. This leads to a negative logistic growth term 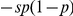 in the equations (10, 12) for the frequency 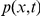 of the mutants. For 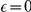, this would trigger a genetic Fisher wave of wild-type invading the mutants. To study the 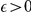 case, observe that the logistic term is carried through all the steps that lead from equation (10) to equation (22). In equation (22), it leads to the replacement



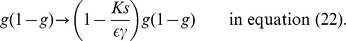



A traveling wave of mutants invading the wild-type population will occur only if this growth term is positive. In other words, the stability condition for a trade-off between growth rate and yield is given by 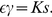
(23)


This criterion was used in the main text to derive the evolutionary stable strategy in equation (5).

We would like to remark that, in contrast to the wave speed, the statement in equation (5) is *independent* of the control parameter 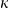. Furthermore, all steps of our analysis, including the nonlinear Cole-Hopf transformation, can be carried out in higher dimensions and result in the same stability criterion as in one dimension. Therefore, the evolutionary stable strategy formulated in equation (5) represents a fairly general result for weak selection.

## Supporting Information

Text S1Detailed analysis of the quasi-static assumption, 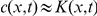, which led to the closed stochastic equation (17) for the mutant frequency.(PDF)
