# Supplementary material for: Noise Driven Evolutionary Waves
Source: PLoS Comput Biol. 2011 Mar 10;7(3):e1002005. doi: 10.1371/journal.pcbi.1002005 (PMC3053316; doi:10.1371/journal.pcbi.1002005)
Supplement: Text S1 — Detailed analysis of the quasi-static assumption, , which led to the closed stochastic equation (17) for the mutant frequency. (PDF) [file pcbi.1002005.s001.pdf]

Noise driven evolutionary waves
Supporting Text S1

Oskar Hallatschek[1,*]
1 Biophysics and Evolutionary Dynamics Group
Max–Planck Institute for Dynamics and Self–Organization
∗ E-mail: oskar.hallatschek@ds.mpg.de

Quasi-static limit.

Here, we analyse the circumstances under which the quasi-static assumption, $c(x,t) \approx K(x,t)$, is justified, which led to the closed stochastic equation (17) of the manuscript for the mutant frequency. To this end, we first describe the full system of stochastic differential equations without approximation, and then discuss the limit for which the density closely follows the carrying capacity.

The stochastic dynamics of the mutant frequency without quasi-static assumption follows from the manuscript's equation (10) by adding the noise term of manuscript's equation (15),

$$\begin{aligned} \partial_t p(x,t) &= D\partial_x^2 p(x,t) + 2D\partial_x \ln c(x,t)\partial_x p(x,t) \\ &+ \eta(x,t)\sqrt{\frac{\gamma p(x,t)[1-p(x,t)]}{K(1+\epsilon p)}} \ . \end{aligned} \tag{1}$$

The noise term in equation (1) corresponds to a branching process with variance γ in the offspring distribution.

The deterministic dynamics of the population density is described by equations (6, 7, 8) of the manuscript. By adding a noise term for the stochastic density fluctuations, we obtain altogether

$$\begin{aligned} \partial_t c(x,t) &= D\partial_x^2 c + c\left[1 - \frac{c}{K(1+\epsilon p)}\right] \\ &+ \eta_c(x,t)\sqrt{\gamma c(x,t)} \end{aligned} \tag{2}$$

The logistic term on the right hand side tends to push the population density towards the carrying capacity corresponding to the local proportion of mutants. The white noise source η_c for density fluctuations is independent on the noise source η for frequency fluctuations.

To reveal the quasi-static limit, we rescale time by the inverse diffusion constant

$$t = \tau/D \ , \tag{3}$$

and substitute

$$c(x,\tau) = K\rho(x,\tau) \ . \tag{4}$$

In terms of ρ and τ, the density equation becomes

$$\partial_\tau \rho(x,\tau) = \partial_x^2 \rho + D^{-1}\rho\left[1 - \frac{\rho}{1+\epsilon p}\right] \tag{5}$$

$$+ \ \eta_c(x,t)\sqrt{\frac{\gamma\rho}{KD}} \ . \tag{6}$$

The equation for the mutant frequency becomes on the other hand

$$\begin{aligned} \partial_\tau p(x,\tau) &= \partial_x^2 p(x,\tau) + 2\partial_x \ln[\rho(x,\tau)]\partial_x p(x,\tau) \\ &+ \eta(x,t)\sqrt{\frac{\gamma p(x,t)[1-p(x,t)]}{KD(1+\epsilon p)}} \ . \end{aligned} \tag{7}$$

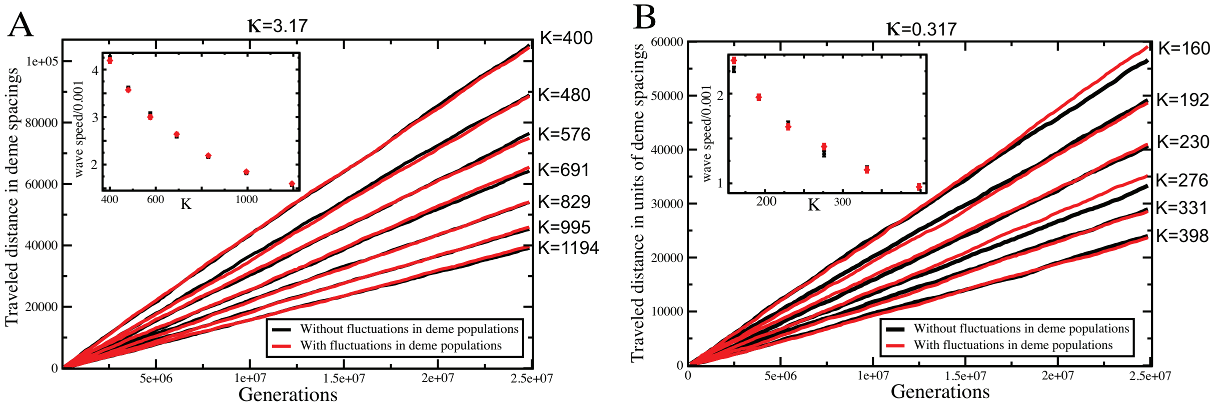

Figure 1. Wave speeds are robust to fluctuations in the deme population. Comparison of our original computer model (red curves) with a "fake" model (black curves), in which the number fluctuations in the total deme population (noise term in Eq. 2) was shut off. The effect of the mutation was set to $\epsilon = 0.1$, and the control parameter $\kappa \equiv KD\epsilon/\gamma$ was chosen to be $\kappa = 0.317$ and $\kappa = 3.17$ for figure A and B, respectively. The main graphs depict time traces of the distance traveled by the wave front versus time for different values of the carrying capacity K. The corresponding wave speeds are depicted in the insets.

Now, consider both equations in the limit

$$D \to 0 \qquad \text{while } KD/\gamma = \text{const.} \,. \tag{8}$$

Notice that the equation for the mutant frequency merely contains the parameter combination KD/γ, such that it remains unaffected by the limit process. However, the density equation (5) simply reduces to

$$\rho(x,t) = 1 + \epsilon p(x,t) \;, \tag{9}$$

because the reaction term $\propto D^{-1}$ dominates over all other terms in (5). Thus, the total density closely follows the carrying capacity on the relevant time scale (of $1/D$). Thus, we can conclude that it is justified to replace ρ in (7) by ϵp in the quasi-static limit defined by equation (8), as was done in the main text. To check that our results for the wave speeds are indeed robust to fluctuations in the deme populations, we simulated a "fake" model, in which the noise term in (2) was shut off. The comparison of the fake and the original model with fluctuations in the deme populations is depicted in Figure 1. We found no significant difference in the results of both simulations for $D \leq 0.1$. For future work, it would be interesting to extend the analysis of the present paper to cases where the quasi-static assumption breaks down.
